# Self-sealing atrio-oesophageal fistula as a complication of pulmonary vein isolation: a case report

**DOI:** 10.1093/ehjcr/ytae283

**Published:** 2024-06-24

**Authors:** Nicolás Herrera-Parra, Enrique Carlos García-Pretelt, Camilo Hernán Bonilla-Cortés, Eduardo Ortíz

**Affiliations:** Departamento de Radiología e Imágenes Diagnosticas, Fundación Valle del Lili, Cra 98 #18-49, 760032 Cali, Colombia; Facultad de Ciencias de la Salud, Universidad Icesi, Cl. 18 #122-135, 760031 Cali, Colombia; Departamento de Radiología e Imágenes Diagnosticas, Fundación Valle del Lili, Cra 98 #18-49, 760032 Cali, Colombia; Facultad de Ciencias de la Salud, Universidad Icesi, Cl. 18 #122-135, 760031 Cali, Colombia; Facultad de Ciencias de la Salud, Universidad Icesi, Cl. 18 #122-135, 760031 Cali, Colombia; Departamento de Radiología e Imágenes Diagnosticas, Fundación Valle del Lili, Cra 98 #18-49, 760032 Cali, Colombia

**Keywords:** Atrio-oesophageal fistula, Pulmonary vein ablation, Stroke, Case report

## Abstract

**Background:**

Atrio-oesophageal fistulas (AEFs) are an uncommon complication of pulmonary vein ablation, and its diagnosis is challenging. Multidisciplinary interventions and diagnostic imaging are usually required and may play a role in the initial assessment.

**Case summary:**

A 69-year-old female with atrial fibrillation who had undergone recent pulmonary vein ablation consulted with unspecific symptoms and sudden hemiparesis. Brain imaging showed pneumocephalus and acute infarcts. Chest computed tomography (CT) was highly suspicious for AEF. Surgical exploration revealed a swollen mediastinum attached to the right inferior pulmonary vein.

**Discussion:**

Non-specific symptoms after pulmonary vein ablation should prompt the suspicion of complications. In the presence of fever or neurological deficit, AEF must be suspected and assessed with a contrast-enhanced chest CT, which has become the gold standard. In brain imaging, pneumocephalus and multiple punctate acute infarcts might also indicate the presence of this complication.

**Learning points**

- Fever and neurological symptoms in patients with recent pulmonary vein ablation should prompt an assessment of atrio-oesophageal fistula (AEF) with contrast-enhanced computed tomography (CT).
- The presence of pneumocephalus in head CT and multiple punctate acute infarcts in brain magnetic resonance imaging might also indicate the presence of this AEF.

## Introduction

Percutaneous left atrial pulmonary vein ablation is a treatment option for rhythm control in patients with atrial fibrillation (AF). It can serve as an alternative to medical therapy, particularly in cases like tachycardiomyopathy.^1^ The incidence of complications following the procedure varies from <1 to 6%, with a meta-analysis reporting a complication rate of 2.9%.^2^ Over time, there have been reports of lower rates, such as 6% in 2005^3^ and 1.5% in 2010,^4^ likely attributed to improvements in technique and technology.

Vascular complications are the most frequent (1.4%), followed by cardiac tamponade (1.0%), pericardial effusion (0.7%), cerebrovascular accident (0.6%), and pulmonary artery stenosis (0.5%). The overall procedure-related mortality rate is 0.06%. Atrio-oesophageal fistulas (AEFs) are less frequent, with an incidence rate of 0.1%.^2^ However, the incidence of AEFs and other complications has been decreasing.

While AEFs pose significant morbidity, they are rare, and their symptoms are non-specific. Therefore, diagnostic imaging plays a crucial role in their identification.

## Summary figure

**Table:** 

8 August 2021	A 63-year-old female with a history of atrial fibrillation was admitted to the emergency room due to cough, asthenia and adynamia, dizziness, diffuse abdominal pain, and fever. She had undergone pulmonary vein isolation 20 days previously. During admission, she suddenly presented with left hemiparesis and dysphasia, following which the stroke code was activated and head computed tomography (CT) performed without any pathological findings.
9–11 August 2021	Leucocytosis (13 900), elevated C-Reactive Protein (CRP) (13.25 mg/dL), and increased lactic acid were documented. Blood cultures reported bacteraemia due to *Streptococcus mitis*, and high-spectrum antibiotics were administered. A simple chest CT and an abdominal CT showed no alterations. Transoesophageal echocardiogram did not reveal vegetations or morphological abnormalities that would explain symptoms. Upper digestive tract endoscopy reported a normal oesophagus throughout its entire length.
16–17 August 2021	She presented with a new fluctuating focal deficit (abnormal movements). Brain magnetic resonance imaging showed two foci of cortical restriction, vermian and right frontoparietal region, which were taken as cytotoxic oedema related to epilepsy. Contrast-enhanced chest CT was taken, showing a filling defect with an air bubble in the left atrium with communication to the oesophagus, findings that were better characterized with a heart CT.
18 August 2021	Surgical exploration revealed a swollen mediastinum attached to the right inferior pulmonary vein. Perforation could not be demonstrated; nevertheless, the site was patched with an autologous intercostal muscle.
29 November 2021	During clinical follow-up, hemiparesis and dysphagia persisted. However, no new symptoms or complications were reported.

## Case presentation

**Figure 1:**
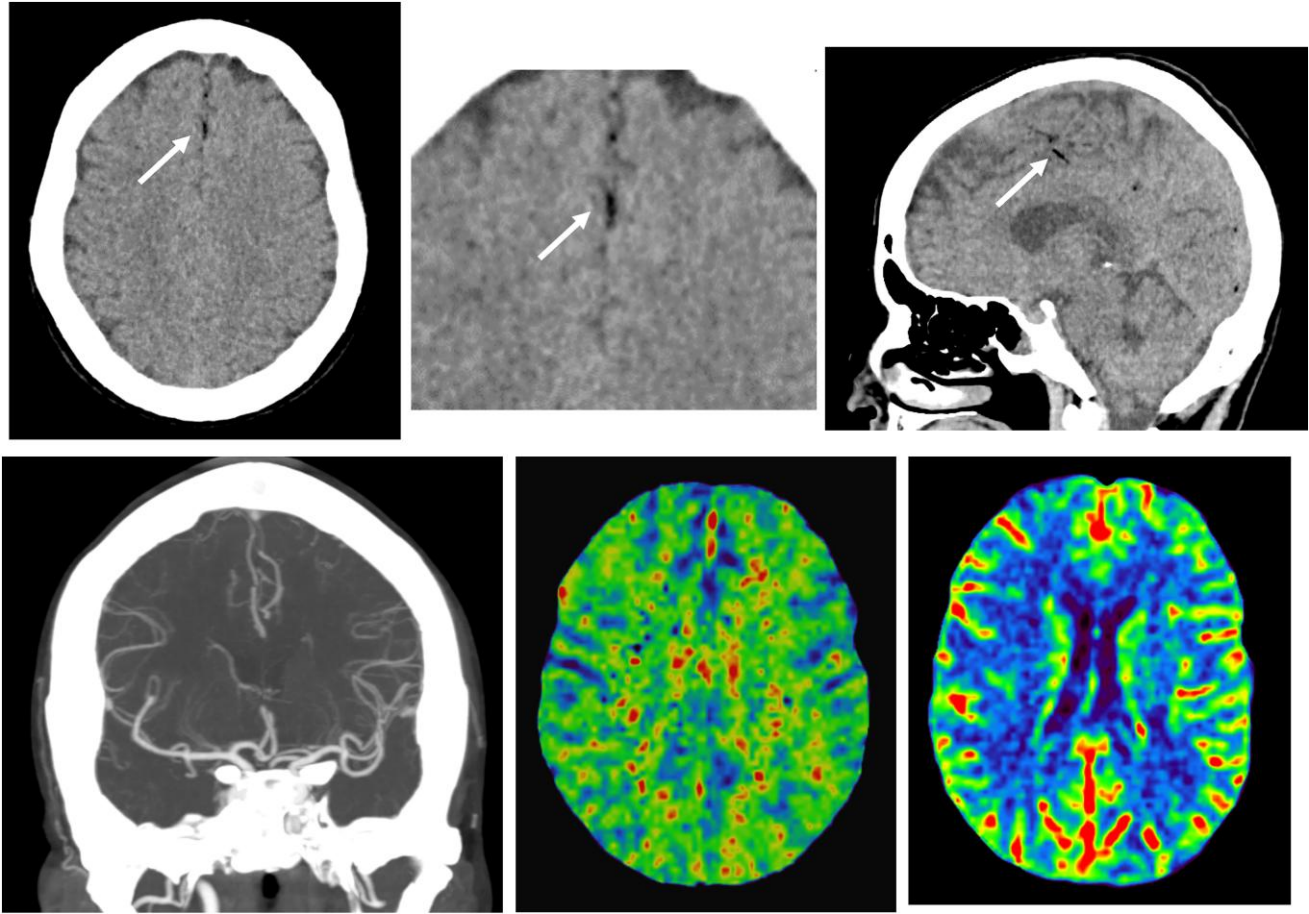
(Above) Head computed tomography. There are no signs of acute ischaemia. Tiny hypodense air bubbles corresponding to pneumocephalus (arrows) are seen in the midline and were omitted in the initial interpretation of the study. (Below) Normal computed tomography brain angiogram and perfusion maps (mean transit time [MTT] and relative cerebral blood volume [rCBV]).

**Figure 2:**
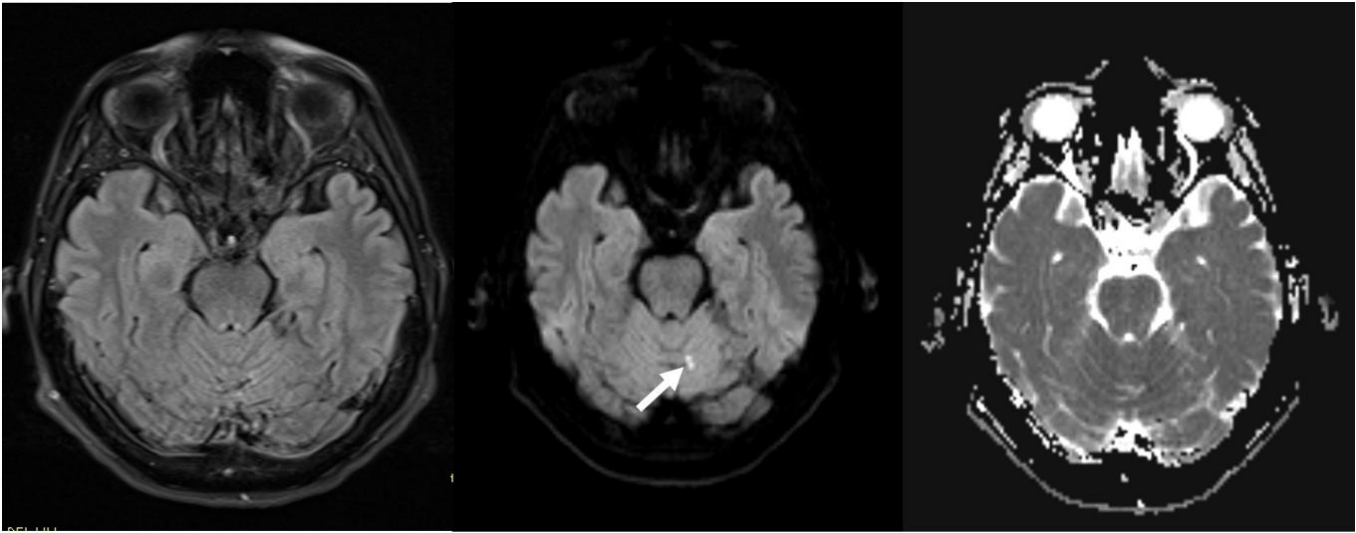
Right to left: Fluid-attenuated inversion recovery (FLAIR T2), diffusion-weighted imaging (DWI), apparent diffusion coefficient (ADC). Diffusion-restricted focus in the left paravermian region of the cerebellum, also seen on FLAIR sequence; there was another similar one in the left frontoparietal cortex (not shown). These findings were consistent with acute lacunar infarcts.

A 63-year-old female with a history of AF presented to the emergency room with cough, asthenia and adynamia, dizziness, diffuse abdominal pain, and fever. The patient had undergone pulmonary vein isolation 20 days previously on an outpatient basis. During admission, the patient experienced left hemiparesis and dysphagia. Head computed tomography (CT) was initially interpreted as normal, but a subsequent examination revealed minimal pneumocephalus (*Figure 1*). Brain magnetic resonance imaging (MRI) indicated two unspecific foci of cytotoxic oedema (*Figure 2*), interpreted as acute lacunar infarcts or epilepsy-related findings.

**Figure 3:**
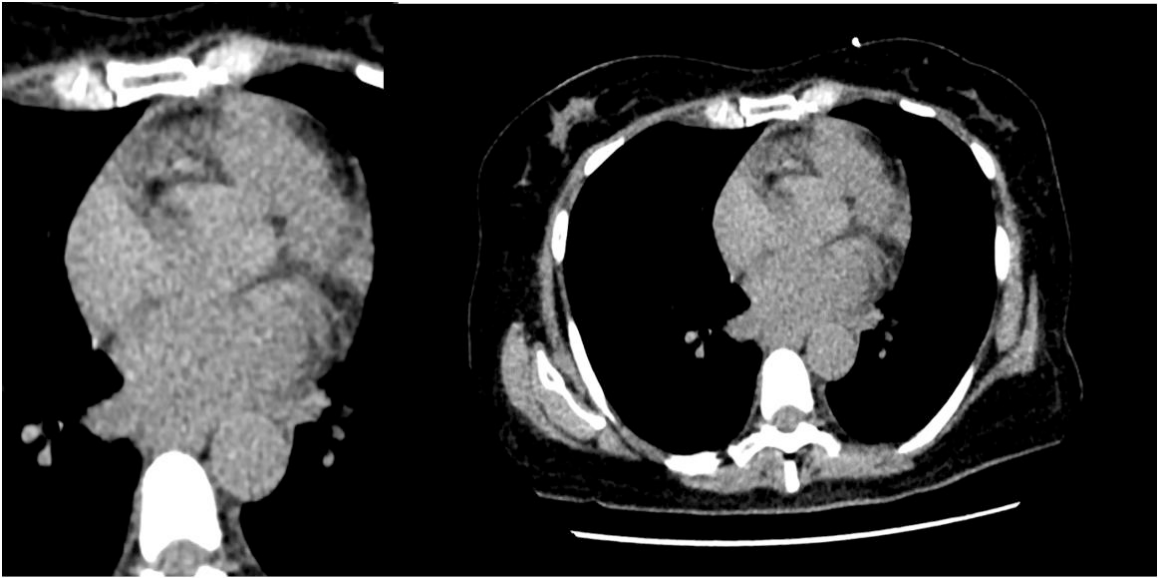
Initial simple chest computed tomography. There are no demonstrable alterations in the mediastinum, changes in the peri-oesophageal fat, pericardial effusion, or pneumopericardium.

The patient presented with leucocytosis (13 900), elevated CRP (13.25 mg/dL), and increased lactic acid. Blood cultures reported bacteraemia (*Streptococcus mitis*), and broad-spectrum antibiotics were administered. A simple chest CT was conducted to investigate pulmonary opacities that were not visualized on the X-ray; it showed normal findings (*Figure 3*). Transoesophageal echocardiogram did not reveal vegetations or morphological abnormalities. Upper digestive tract endoscopy was performed due to non-specific gastrointestinal symptoms, with no suspicion of AEF at the time; it reported a normal oesophagus throughout its entire length.

**Figure 4:**
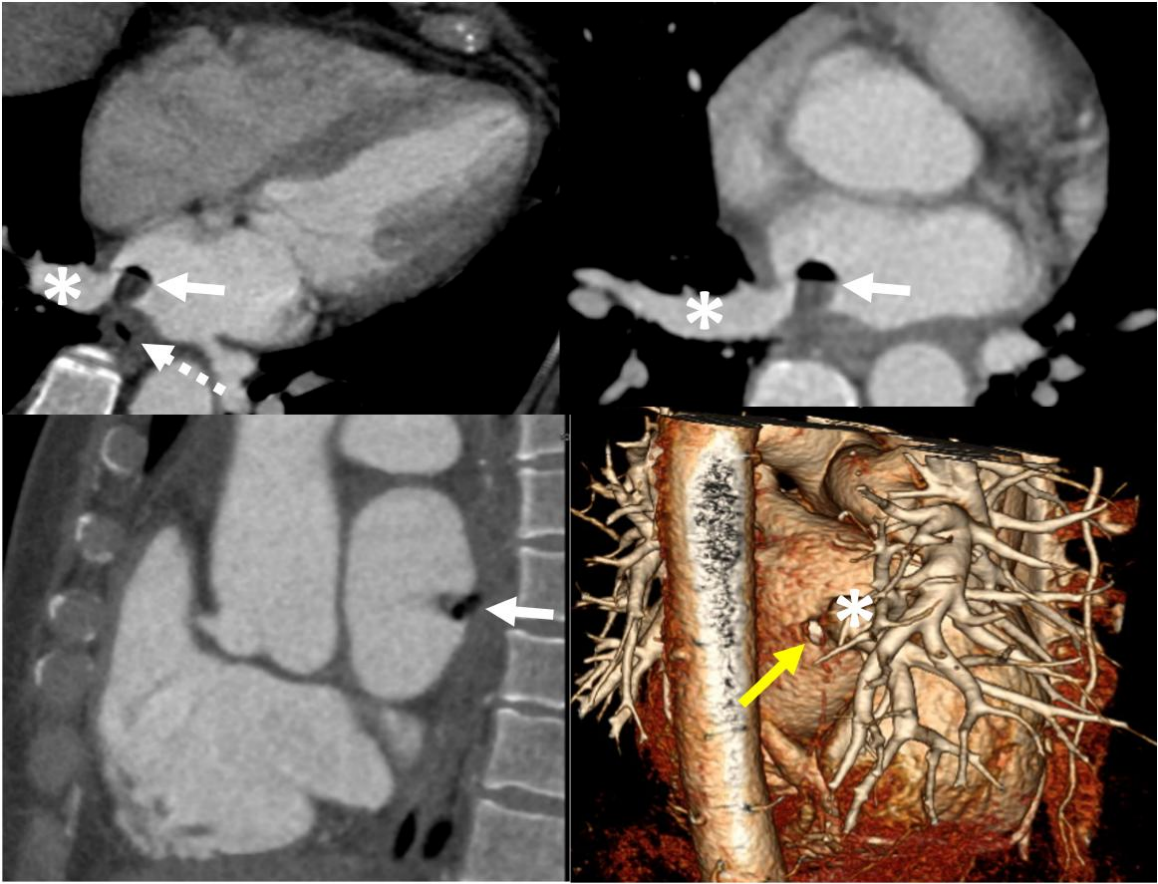
(Above) Contrast-enhanced axial chest computed tomography showing a filling defect in the left atrium with a small air bubble (white arrow) located at the base of the right inferior pulmonary vein (asterisk) with a fistulous tract connecting this filling defect to the oesophagus (dashed arrow). (Below left) A gated sagittal heart shows the same findings. (Below right) A volume-rendered three-dimensional image of the heart depicts the fistula in the posterior left atrium wall (yellow arrow), adjacent to the right inferior pulmonary vein (asterisk).

Eight days after admission, contrast-enhanced chest CT was performed due to the emergence of suspicion about an AEF. The image demonstrated a filling defect with an air bubble in the left atrium. This finding was further characterized using gated heart CT, demonstrating AEF (*Figure 4*).

Surgical exploration was performed 11 days after admission, and it revealed a swollen mediastinum attached to the posterior wall of the left atrium, adjacent to the right inferior pulmonary vein. Although perforation could not be demonstrated, the site was patched with an autologous intercostal muscle.

The patient was discharged and followed up at 3, 6, and 12 months. She presented with a complete alleviation of symptoms. No new symptoms or complications were reported.

## Discussion

The natural history of AEF following radiofrequency ablation (RFA) is unknown. It is believed that direct thermal damage to the oesophageal mucosa, along with predisposing factors such as gastro-oesophageal reflux disease and reduced oesophageal motility (due to nerve damage or general anaesthesia), induces inflammation, ulceration of the oesophageal mucosa, and eventual perforation. Cases at intermediate stages have been reported.^5^ Vascular damage to the anterior oesophageal arteries may also play a role, explaining their late appearance in some cases.^5^ In a single-centre study, where 800 patients underwent endoscopy after pulmonary vein ablation, oesophageal erythema/erosion was found in 12%, ulcers in 6%, perforation in 0.4%, and atrio-oesophageal or oesophagopericardial fistulas in 0.2%.^6^

Risk factors for thermal damage to the oesophagus include the use of general anaesthesia, nasogastric tubes, high levels of radiofrequency power (>25–30 W), non-irrigated catheters, left atrial enlargement, low body mass index, high temperatures during ablation (an increase of 1.5–2°C or >38.5–42°C), the number of ablative lesions in the posterior wall of the left atrium, and gastro-oesophageal reflux disease, among others.^7^ Furthermore, the POTTER AF study reported a significant higher incidence of AF after RFA (0.038%) than after cryoballoon (0.0015%; *P* < 0.0001),^8^ depicting that the type of procedure should be taken into account, but it did not exclude the possibility of this complication. Our patient received RFA using fixed energy levels of 25 W at the posterior wall without an oesophageal probe; nevertheless, it has been observed that intra-oesophageal temperature monitoring does not affect the incidence of endoscopically diagnosed oesophageal lesions^9^.

The typical location for AEFs following RFA ablation is near the left inferior pulmonary vein due to spatial constraints between the atrium and the oesophagus in this area.^5^ Moreover, the temperature changes in the oesophagus are most pronounced in this area. However, in our patient, the fistula was found adjacent to the right inferior pulmonary vein, deviating from the expected pattern.

Our patient’s clinical presentation aligns with the reported symptoms associated with AEFs. Fever (73%) and neurological manifestations such as focal deficits and seizures (72%) are prevalent, while gastrointestinal symptoms, including melena or dysphagia, may also manifest (present in <50% of patients). Additionally, chest pain often serves as a prodromal symptom (43%). Consistent with existing literature, these symptoms typically emerge around 21 days post-ablation.^7^

Oesophageal endoscopy has a low yield (13.7%)^10^ and, if suspected, is contraindicated due to the potential to trigger embolic events in the central nervous system.^11^ In our patient, oesophageal endoscopy was performed due to non-specific gastrointestinal symptoms; however, at the time of examination, there was no suspicion of an AEF. Consequently, the endoscopic findings were normal. This underscores the challenge of relying solely on endoscopic evaluation.

Contrast-enhanced tomography is the preferred imaging modality and the gold standard for diagnosing AEFs. It can diagnose up to 65% of cases, with normal findings in up to 25% of patients (those in the early stages of the disease). Repeating the study can help achieve a diagnosis in practically all patients.^10,11^ Conclusive imaging signs include the presence of air in the left atrium, mediastinum, or pericardium, contrast medium extravasation into the oesophagus, or clear demonstration of the fistula.^10,12^

In patients in whom the chest scan appears normal, pathological findings in brain CT or MRI can help clarify the diagnosis. Computed tomography abnormalities are found in 51% of patients, while MRI abnormalities are found in 87%.^10,11^ The most frequent findings include diffuse air embolism or ischaemia (79%) and focal air embolism or focal ischaemia (18%). In our patient, a review of the simple head CT and brain MRI images revealed pneumocephalus and acute lacunar infarcts, which were initially overlooked and could have provided some guidance regarding the patient’s condition, leading to an earlier diagnosis.

Differential diagnoses based on imaging include a spectrum of similar lesions such as oesophagitis and ulcers (manifesting as a thickening of the oesophageal walls on CT), oesophageal perforation (resulting from direct damage or entities like Mallory–Weiss laceration or Boerhaave syndrome, leading to pneumomediastinum or peri-oesophageal fluid), and oesophagopericardial fistula (manifesting as haemo or pneumopericardium).^7,13^

Treatment options for fistulas include conservative management, endoscopic intervention with oesophageal stenting, or surgical intervention. Conservative management carries the highest mortality rate (90%), followed by endoscopic intervention (65%) and surgical intervention (33%).^6,11^ Complications are frequent, and the mortality rate for this condition ranges from 55 to 67%, with central nervous system complications being the most common.^7,13^

In our patient, the surgical approach (carried out 11 days after admission) revealed an inflamed mediastinum attached to the posterior wall of the left atrium, without identifying perforations. There are no reports in the literature of ‘self-sealed’ AEFs; however, it is possible that this occurred in our patient. The intraoperative decision was to seal the site with an autologous intercostal muscle graft, with favourable post-operative outcomes.

## Conclusion

In summary, oesophageal–pericardial fistulas are rare but concerning complications of pulmonary vein ablation, necessitating prompt radiological diagnosis. Contrast chest CT is the most critical imaging modality. If the initial scan yields normal results but suspicion remains high, repeating the study can aid in achieving a diagnosis. Abnormal brain imaging findings can also support the diagnosis within the appropriate clinical context.

## Data Availability

The relevant anonymized patient-level data are available upon request from the authors.

## Lead author biography

Nicolas Herrera Parra is a 4th year radiology resident from Bogotá, Colombia. He is a radiology resident at Universidad Icesi, Fundación Valle del Lili in Cali, Colombia. He completed his medicine studies at Universidad Javeriana. He loves trekking, music, and radiology.

## Notes

**Consent:** The authors confirm that written consent for submission and publication of this case report, including image(s) and associated text, has been obtained from the patient in line with COPE guidance. An individual patient informed consent form was signed.

**Funding:** The authors received no financial support for the research, authorship, and/or publication of this article.

**Ethical approval:** The reported case was reviewed and approved by the institutional ethics committee. Following their guidelines, all protected health information were removed.
